# Neonatal Noonan syndrome with acute kidney injury and systemic capillary leak syndrome caused by a *RIT1* variant: a case report and literature review

**DOI:** 10.3389/fped.2026.1838907

**Published:** 2026-07-09

**Authors:** Chunfang Gao, Fanli Kong, Xuwei Tao

**Affiliations:** 1 Division of Neonatology, Wuhan Children's Hospital (Wuhan Maternal and Child Healthcare Hospital), Tongji Medical College, Huazhong University of Science & Technology, Wuhan, China; 2 Pain and Sleep Treatment Center, Wuhan Children's Hospital (Wuhan Maternal and Child Healthcare Hospital), Tongji Medical College, Huazhong University of Science & Technology, Wuhan, China

**Keywords:** acute kidney injury, Noonan syndrome, RAS-MAPK pathway, *RIT1* gene, systemic capillary leak syndrome

## Abstract

**Objective:**

To report a rare case of neonatal Noonan syndrome (NS) caused by a *RIT1* c.247A>C (p.Thr83Pro), and to expand the understanding of life-threatening presentations in neonatal *RIT1* associated NS.

**Methods:**

The clinical data of a neonate with NS admitted to the Neonatal Intensive Care Unit of Wuhan Children's Hospital were retrospectively analyzed, and *Vitro* functional studies were performed to investigate the pathogenic mechanism of the variant. A literature review of *RIT1*-NS cases reported between 2014 and 2025 was also performed.

**Results:**

The proband was a term female neonate presenting with progressive acute kidney injury (AKI), systemic capillary leak syndrome (SCLS), refractory chylothorax, and a large patent ductus arteriosus. Whole-exome sequencing identified a *de novo* heterozygous *RIT1:*c.247A>C p.(Thr83Pro). Functional studies revealed that this variant significantly enhanced the phosphorylation of ERK, JNK, and p38, and markedly upregulated IL-1β, IL-6, and TNF-α mRNA expression. Among 54 reported *RIT1*-NS cases, the p. Thr83Pro variant accounted for 3.7% (2/54); our case was the only one presenting with AKI and SCLS. Among the reported neonatal cases, 4 of 10 died during the neonatal period.

**Conclusion:**

The *RIT1:*c.247A>C p.(Thr83Pro) might contribute to life-threatening NS in the neonatal period, and that early genetic testing may facilitate diagnosis and prognostic assessment.

## 1 Introduction

Noonan syndrome (NS, MIM 163950) is an autosomal dominant disorder and the most common subtype within the RASopathies, with an estimated birth incidence of 1 in 1,000 to 1 in 2,500 live births (1). Its core pathogenesis involves aberrant hyperactivation of the RAS-MAPK signaling pathway, leading to dysregulated cellular proliferation, differentiation, and apoptosis (2, 3). Clinically, NS presents with a broad spectrum of manifestations, most notably lymphatic dysplasia and congenital heart disease, and may also be accompanied by coagulation disorders, growth retardation, intellectual disability, and ectodermal abnormalities (4, 5). Since its identification as a novel NS-associated gene in 2013, variants in *RIT1* (MIM 609591) have been reported to account for approximately 5% of genetically confirmed NS cases (6, 7). Patients with *RIT1* variants typically present with a classic phenotype, frequently characterized by high birth weight, perinatal lymphatic anomalies (including hydrops fetalis, increased nuchal translucency, and polyhydramnios), and cardiovascular defects. However, the genotype–phenotype correlation of *RIT1*associated NS (*RIT1*-NS) remains to be fully elucidated (8). Here, we report a neonate admitted to Wuhan Children's Hospital in April 2025 who presented with progressive acute kidney injury (AKI), systemic capillary leak syndrome (SCLS), chylothorax, and a large patent ductus arteriosus (PDA), and was subsequently diagnosed with *RIT1*-NS. Through *in vitro* functional assays, we obtained supportive evidence for the potential pathogenicity of the variant, which may contribute to expanding the genotype-phenotype spectrum of neonatal NS.

## 2 Material and methods

### 2.1 Clinical data collection

Clinical data were collected from a female neonate with a confirmed genetic diagnosis. Information regarding prenatal history, perinatal events, physical examination findings, laboratory tests, imaging studies, and clinical course were retrospectively reviewed.

### 2.2 Whole-exome sequencing and genetic analysis

Genomic DNA was extracted from peripheral blood samples of the proband and her parents. Whole-exome sequencing (WES) was performed to identify potential pathogenic variants. Candidate variants were filtered and annotated using standard bioinformatics pipelines. Sanger sequencing was subsequently used to validate the identified variant and confirm its inheritance pattern. Variant classification followed the American College of Medical Genetics and Genomics (ACMG) guidelines (9).

### 2.3 *In vitro* functional validation

**Figure 1:**
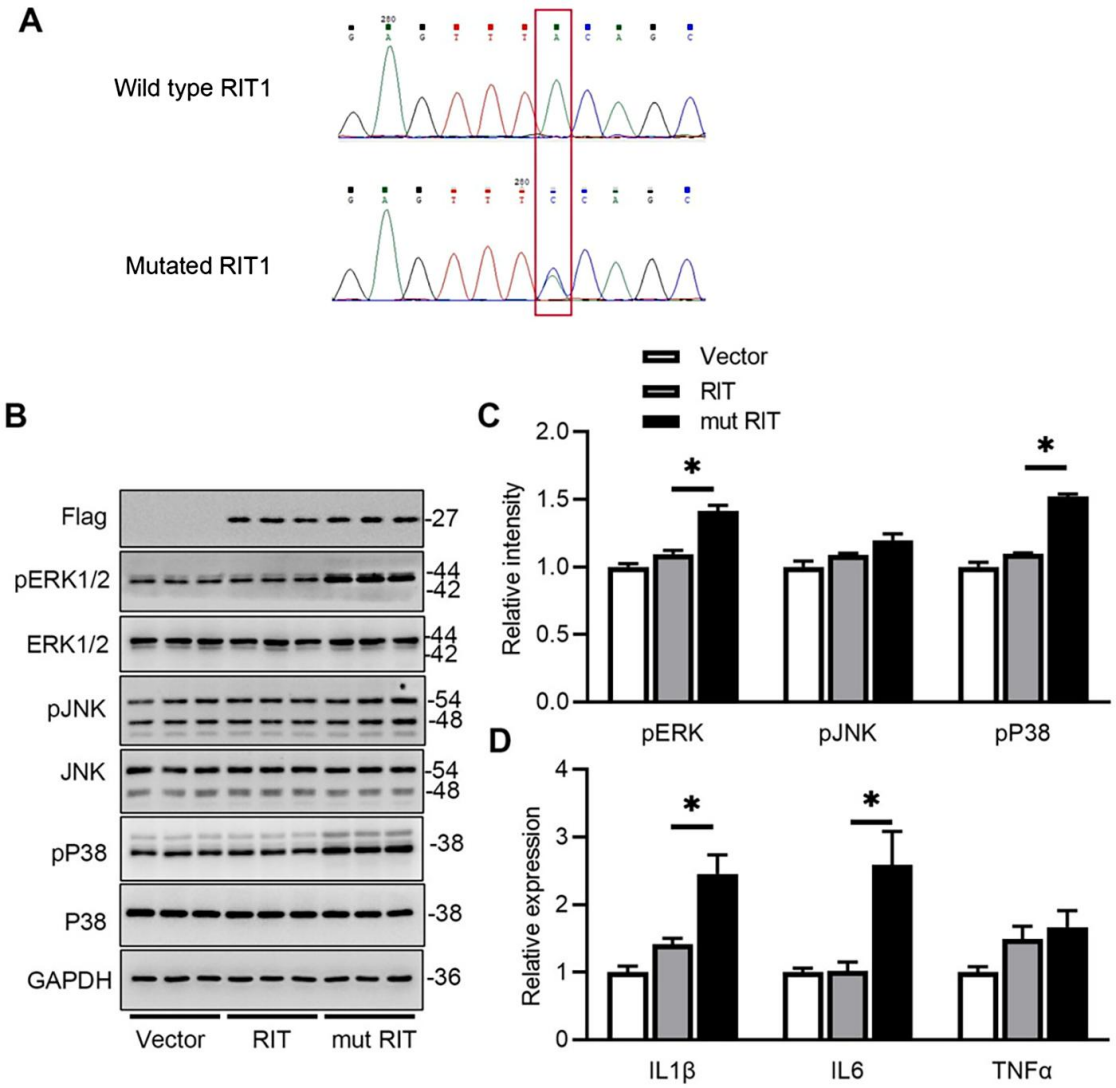
Mutated *RIT1* activated the MAPK pathway. **(A)** Wild type/mutated *RIT1* (c.247A>C) was constructed into pcDNA 3.1 vector; **(B,C)** Over-expressing wild type/mutated *RIT1* in RLE-6TN wt cells for 48 h, and examined the MAPK pathway using Western blot and its statistical graph; **(D)** The mRNA level of IL-1β, IL-6, TNF-α using qPCR. One-way ANOVA, *P < 0.05.

To investigate the functional impact of the identified *RIT1* variant, plasmids carrying wild-type *RIT1* (*Wt*-*RIT1*) and the mutant *RIT1* (*Mut*-*RIT1*) were constructed (Beijing Aoke Biotechnology Co., Ltd) (Figure 1), and transfected into rat alveolar type II epithelial cells (RLE-6TN) using Lipofectamine 3,000 (Thermo Fisher Scientific) according to the manufacturer's instructions. Following transfection, cells were cultured in fresh DMEM medium for 48 h, after which total protein was extracted. Proteins were separated by SDS-PAGE and transferred to nitrocellulose membranes, followed by incubation with primary antibodies against ERK1/2, JNK, p38, and their phosphorylated forms (p-ERK1/2, p-JNK, p-p38; CST, USA). GAPDH (Proteintech, Wuhan, China) served as the loading control. Membranes were then incubated with horseradish peroxidase-conjugated secondary antibodies (1:1,000; Beyotime, Shanghai, China). Protein bands were visualized using ECL chemiluminescence reagents (Biosharp, Beijing, China), and densitometric analysis was performed using ImageJ software (National Institutes of Health, USA). For mRNA expression analysis, quantitative PCR (qPCR) was performed using the following primers: IL1-β-F GACTTCACCATGGAACCCGT, IL-1β-R GGAGACTGCCCATTCTCGAC; IL6-F AGCGATGATGCACTGTCAGA, IL-6-R GGAACTCCAGAAGACCAGAGC; TNF-α-F CATCCGTTCTCTACCCAGCC, TNF-α-R AATTCTGAGCCCGGAGTTGG; ACTB-F CGATATCGCTGCGCTCGT, ACTB-R ATACCCACCATCACACCCTG. All data are presented as mean ± standard deviation (SD). Statistical significance was assessed by one-way ANOVA using GraphPad Prism 8, with a *P* value <0.05 considered statistically significant.

### 2.4 Literature review

We searched PubMed, Web of Science, and the China National Knowledge Infrastructure (CNKI) for case reports of *RIT1*-associated Noonan syndrome published from 2014 to 2025. A total of 54 patients (including the present case) were identified for analysis (10–20). Genotype and phenotype data were extracted and summarized.

## 3 Results

### 3.1 Clinical presentation and course

**Figure 2:**
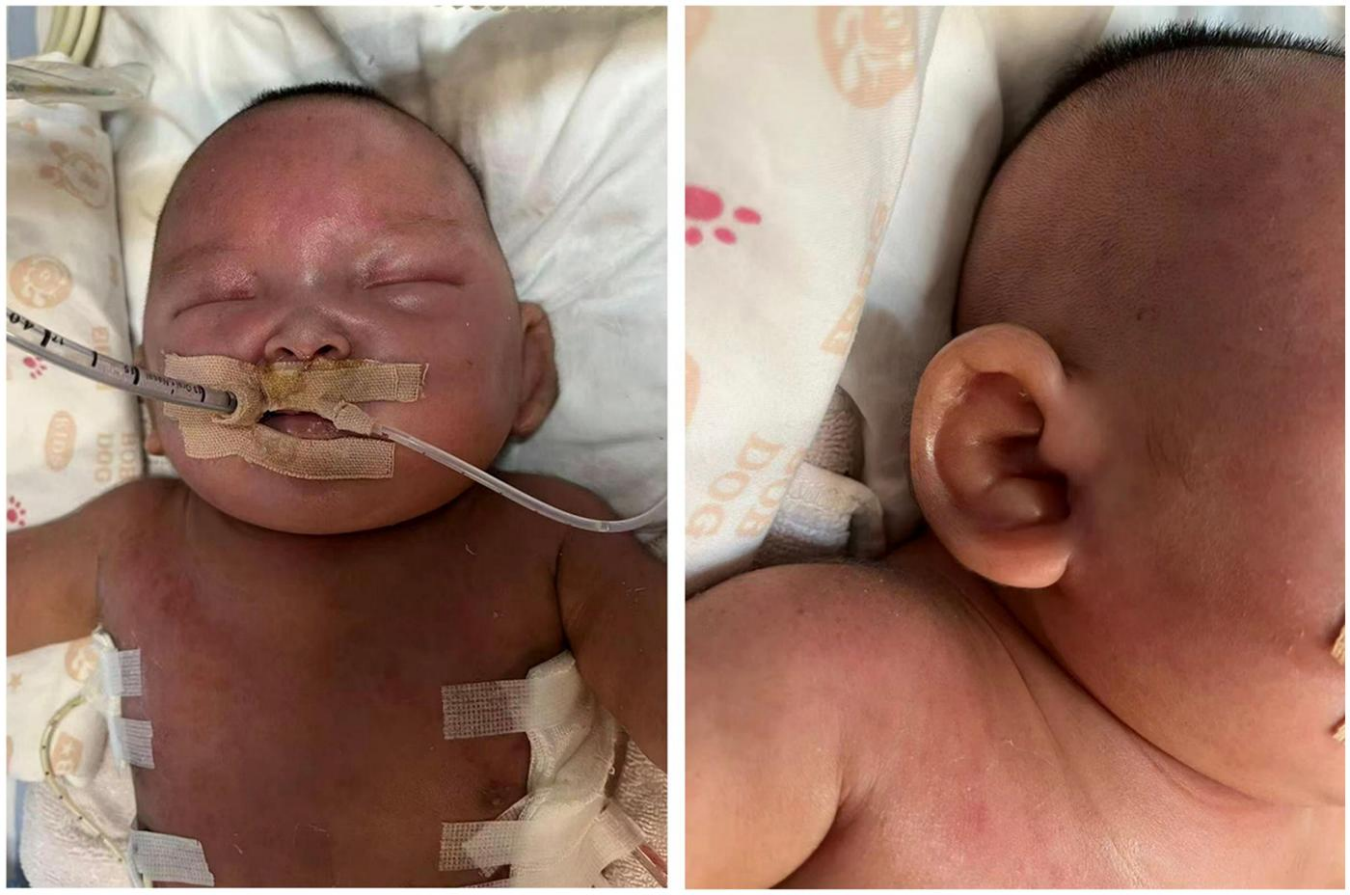
The patient's facial dysmorphism.

The proband was a G4P2 female neonate delivered by cesarean section at 37′4 weeks of gestation. Birth weight was 4.37 kg (>97th percentile, +3.8 SD), head circumference 35 cm (97th percentile, +1.5 SD), and length 51 cm (>97th percentile, +1.9 SD). Apgar scores were 9 at both 1 and 5 min. The placenta and umbilical cord appeared normal. Prenatal ultrasound revealed polyhydramnios (amniotic fluid index >35 cm). Physical examination at birth revealed a depressed nasal bridge, low-set ears, and a shortened neck; no other remarkable dysmorphic features were observed (Figure 2). The mother, aged 42 years, had a history of gestational diabetes mellitus and hypothyroidism during pregnancy, both well controlled with insulin and levothyroxine. The father and an elder sister were healthy, with no family history of hereditary metabolic disorders.

On day 3 after birth, the infant developed poor responsiveness and feeding difficulties without an obvious precipitating cause, followed by respiratory distress. Ultrasound revealed bilateral pleural effusion, with a daily drainage volume exceeding 20 mL/kg; chylous effusion was confirmed by positive chyle test. A distinct precordial murmur was noted, and echocardiography demonstrated a patent ductus arteriosus (PDA) measuring 6.4 mm with bidirectional shunting. During the first 3 days of life, routine blood tests, biochemistry, and electrolytes were all within normal limits; only coagulation revealed mild hypofibrinogenemia, with a nadir of 1.01 g/L (reference range: 1.4–4.0 g/L). On day 5, the infant developed bilateral lower extremity edema that rapidly progressed to generalized edema, accompanied by hypotension, with the laboratory findings showed hypoalbuminemia, hemoconcentration (hematocrit 65%), and electrolyte disturbances (hyponatremia, hypocalcemia), meeting the diagnostic criteria for SCLS. In addition, coagulation abnormalities were noted, including prolonged prothrombin time (PT) and activated partial thromboplastin time (APTT), (peak values: PT 30 s, APTT 89 s, with reference of 13 s–20 s, 45 s–65 s) and a significant reduction in fibrinogen to 0.62 g/L. On day 10, the patient developed oliguria that progressed to anuria, with progressively elevated serum creatinine and blood urea nitrogen levels, fulfilling the diagnostic criteria for AKI.

During hospitalization, the infant received mechanical ventilation, crystalloid fluid resuscitation, intermittent infusions of plasma and albumin, vasoactive agents (including dopamine and dobutamine) to support circulatory function, continuous renal replacement therapy, and PDA ligation. Despite these interventions, the patient's condition continued to deteriorate, and she died on day 36 after birth.

### 3.2 Genetic findings

**Figure 3:**
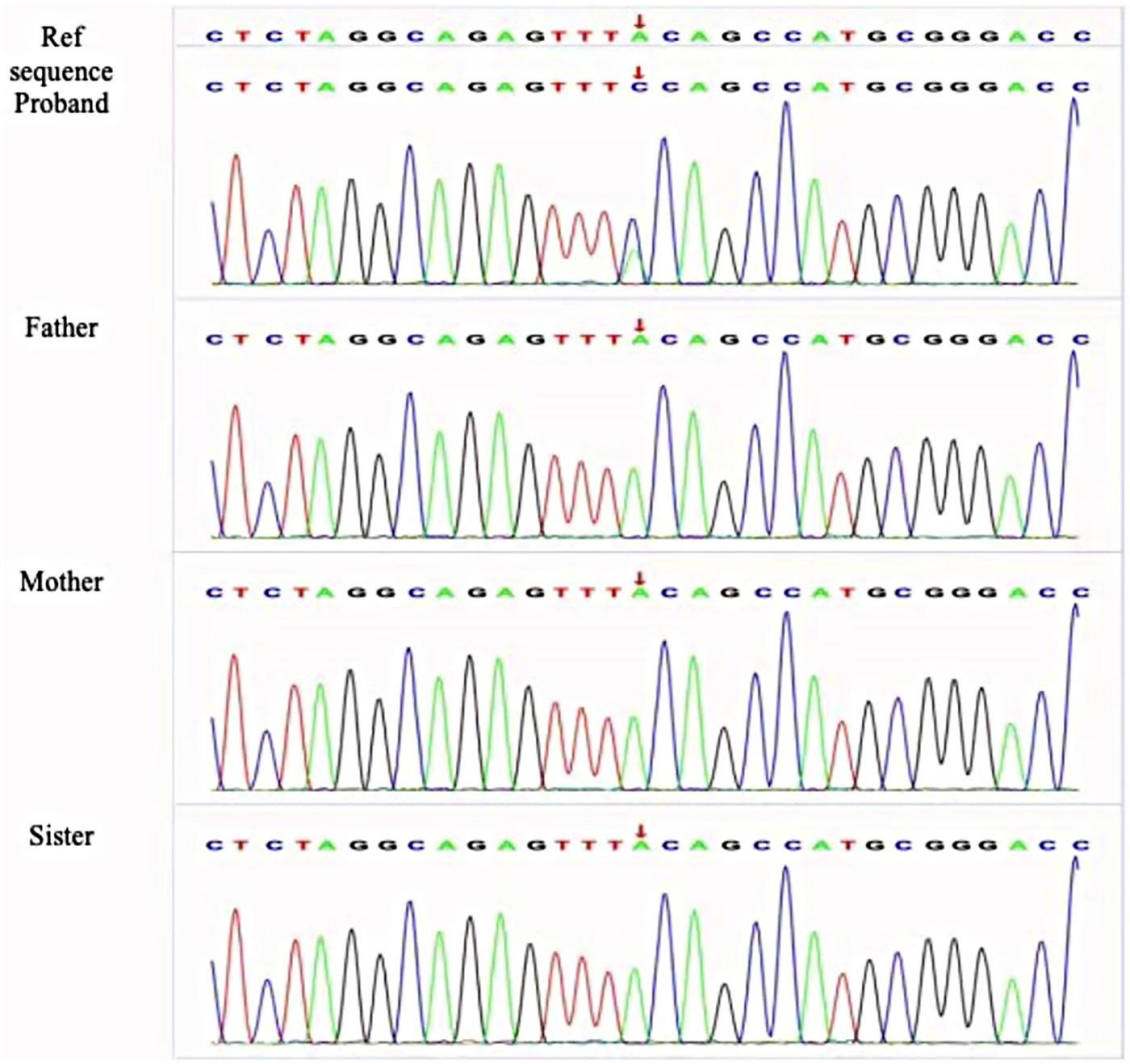
Whole exome sequencing of the family.

WES identified a heterozygous variant in *RIT1* gene [NM_006912.5: c.247A>C (p.Thr83Pro)], which was confirmed as a *de novo* variant in the family (Figure 3). Based on the ACMG (9), this variant was classified as likely pathogenic, supported by the following evidence: confirmed *de novo* (PS2), located in a critical functional domain of RIT1 (PM1), absent from population databases (PM2), and predicted deleterious by multiple in silico tools (PP3).

### 3.3 *In vitro* functional results

Functional assays demonstrated that the p.Thr83Pro variant led to increased phosphorylation of ERK1/2, JNK, and p38 compared to *Wt-RIT1*, indicating enhanced activation of the RAS-MAPK signaling pathway. qPCR analysis further revealed significant upregulation of pro-inflammatory cytokines, including IL-1β, IL-6, and TNF-α, in cells expressing the mutant construct (*P* < 0.05) (Figures 1B–D).

### 3.4 Literature review results

A total of 54 patients (including the present case) were enrolled for analysis (10–20); and the detailed genotype–phenotype were summarized in Table 1. The results showed that *RIT1* variants predominantly clustered in the Switch II functional domain (1), accounting for 68.5% (37/54) of cases, with *p.*Ala57Gly (27.7%), *p.*Gly95Ala (20.3%), and *p.*Phe82Leu (11.1%) being the most common variant types. The p.Thr83Pro variant identified in our case accounted for 3.7% (2/54); the other affected individual was a 2-month-old infant with the same variant who presented with a classic NS phenotype and had a favorable prognosis (13). Among the 54 patients, 10 were diagnosed neonatally, of whom 4 died during the neonatal period, suggesting a poor prognosis for neonatal onset that may be related to the functional impact of the specific variant.

**Table 1:** The genotype–phenotype of *RIT1* associated noonan syndrom**e**.

Case	age	gender	variant	Abnormal prenatal findings (nuchal edema, polyhydramnios, cystic hygroma)	High birth weight	Preterm	Feeding difficulties	CHD	Lymphatic abnormality	Growth retardation	Motor delay	Intellectual disability	Genitourinary abnormality	Craniofacial dysmorphism (ptosis, downslanting palpebral fissures, hypertelorism, low-set ears, short neck)	Ectodermal dysplasia (cutaneous and hair abnormalities: curly hair, sparse eyebrows, sparse eyelashes)	Skeletal abnormality (pectus carinatum, pectus excavatum, scoliosis)	Coagulation abnormality	Tumor	Others	Outcome
Gos, 2014 (*n* = 4) (10)	5.5–17.5y	4F	p.P82V, p.M90I, p.G95A(2)	2/2	3/4	0	4/4	PS(4), HCM(2), PDA(1), VAD/VSD(1)	1/2	4/4	2/4	4/4	ND	4/4	2/4	4/4	1/4	－	－	Alive
Bertola, 2014 (*n* = 6) (11)	2–-28y	4M/2F	p.S35T, p.G95A(2), p.A57G(2), p.F83L	2/2	5/6	1/6	ND	PS(5), HCM(2)，PDA(1)，ASD/VSD(2)	ND	ND	ND	0	Cryptorchidism (3/3)	6/6	5/5	3/6	2/6	－	SLE, Dandy–Walker anomaly, Graves disease	ALive
Koenighofer, 2016 (*n* = 2) (12)	2–15y	M/F	p.M90I, p.A57G	2/2	2/2	1/2	1/2	PS(1), HCM(2), PDA(1)，ASD/VSD (2)	1/2	－	ND	ND	ND	2/2	1/2	1/2	ND	－	－	ALive
Kouz,et al. 2016(*n* = 33) (13)	2m–16y	11M/22F	p.A57G(9), p.G95A(6), p.F82L(6), p.F82 V(1), p.M90I(1), p.S35T(3), p.G31R(2), p.K23N(1), p.T83P(1), p.A77T(2), p.F82S(1)	15/29	ND	15/29	13/27	PS(26), HCM(14)，ASD/VSD(12)	13/30	20/30	9/29	6/22	Cryptorchidism(6/11), Hydronephrosis(3), Mild Renal dysfunction(4)	26/33	13/33	16/33	6/29	Multiple giant cell tumor of the jaw (1)，B-cell acute lymphoblastic leukemia (1)，B-cell acute lymphoblastic leukemia (1)，Lipoma (1)	MDD, Hydrocephalus	Alive
Ramond, 2017(*n* = 1) (14)	3y	M	p.G81G	－	–	–	＋	LMCA atresia	－	＋	－	＋	－	＋	＋	＋	－	－	Sigmoid stricture	Dead
Chen, 2019 (*n* = 4) (16)	2.4–17m	3F/M	p.A57G(3), p.M90I(1)	ND	ND	ND	ND	PS(4), HCM(4), PDA(1)，ASD/VSD(4)	ND	ND	ND	ND	ND	ND	ND	ND	ND	－	－	Alive
Safwat,2020 (*n* = 1) (15)	4d	M	p.A74G	＋	＋	－	ND	PS	－	ND	ND	ND	ND	－	－	－	ND	－	Mononucleosis	Dead
Zha, 2022(*n* = 1) (18)	3d	M	p.A57G	＋	ND	－	＋	ASD/VSD	＋	＋	－	－	Cryptorchidism	＋	＋	ND	－	－	－	Alive
Kyogo,2023 (*n* = 1) (17)	14d	F	p.M90I	＋	ND	－	ND	PS, ASD/VSD	＋	ND	ND	ND	ND	＋	－	ND	ND	MPN		Dead
Claudia, 2023(*n* = 1) (19)	2m	F	*	＋	ND	＋	ND	PS, ASD/VSD, Coronary artery ectasia	－	ND	ND	ND	－	＋	－	ND	ND	－	－	Alive
Yuka, 2025 (*n* = 1) (20)	8.5y	F	p.G95A	ND	＋	＋	ND	–	＋	ND	ND	ND	Genital abnormality	＋	－	－	ND	－	－	Alive
our case, 2025 (*n* − 1)	2d	F	p.T83P	＋	＋	－	＋	PDA，ASD/VSD	＋	ND	ND	ND	AKI	＋	－	－	－	－	SCLS	Dead

ND, Not defined; M, male; F, Female; CHD, Congenital heart disease; PS, Pulmonary stenosis; HCM; hypertrophic cardiomyopathy; VSD, Ventricular Septal Defect; VSD, Ventricular Septal Defect; ASD, Atrial septal defect; LMCA atresia, Left main coronary artery atresia; AKI, Acute kidney injury; SLE, systemic lupus erythematosus; MDD, Major depressive disorder; MPN, Myeloproliferative neoplasms; SCLS, Systemic capillary leak syndrome.

Regarding clinical manifestations, cardiovascular defects were the most frequent finding, occurring in 96.2% (52/54) of patients (21), predominantly pulmonary stenosis (PS, 83.3%, 40/48), hypertrophic cardiomyopathy (HCM, 51.1%, 24/47), and atrial or ventricular septal defects (ASD, VSD,48.8%, 21/43), while patent ductus arteriosus was observed in 8.9% (PDA, 4/45). Lymphatic system abnormalities were reported in 51.4% (18/35). Renal abnormalities were mostly represented by hydronephrosis (3cases); severe AKI was reported only in our case, as was SCLS. In addition, high birth weight (>90th percentile) was observed in 86% (13/15) of patients with *RIT1*-NS, a frequency significantly higher than that in other subtypes of NS and may serve as a suggestive phenotype for *RIT1* variants.

## 4 Discussion and limitations

The *RIT1* gene, located on the long arm of chromosome 1, encodes a small GTPase that regulates RAS-MAPK pathway activity through guanine nucleotide binding and hydrolysis via conserved G-box motifs (22). Vascular endothelial growth factor receptor 3 (VEGFR3) is a key regulator of lymphatic development, promoting lymphatic endothelial cell proliferation, migration, and tube formation through downstream signaling cascades including RAS-MAPK and PI3K-AKT; disruption of VEGFR3 signaling is a well-established mechanism underlying various lymphatic dysplasias (23). Gain-of-function variants in *RIT1* lead to aberrant RAS-MAPK hyperactivation, which can transcriptionally modulate VEGFR3 expression (23), interfere with its downstream effector signaling (24), and ultimately impair VEGFR3-mediated pro-lymphangiogenic pathways (25). This concurrent state of RAS-MAPK pathway hyperactivation and impaired VEGFR3 downstream signaling may represent the core molecular mechanism underlying lymphedema, a typical clinical feature of Noonan syndrome (26, 27).

In this case, the neonate with NS carried a *de novo RIT1* c.247A>C variant and exhibited characteristic features of *NS*, including high birth weight, chylothorax, and a large PDA. Notably, the patient also developed progressive AKI and SCLS, manifestations not previously reported, thereby expanding the clinical phenotype of the disease. The variant is located in the Switch II domain of the RIT1 protein, a highly conserved region critical for GTPase activity and a known hotspot for pathogenic NS variants. Variants in this region enhance downstream RAS-MAPK signaling and reduce proteasomal degradation, leading to protein accumulation and consequent signal hyperactivation (22, 28). *In vitro* functional validation confirmed that this variant significantly increases the phosphorylation levels of ERK and p38 within the MAPK pathway and promotes the release of the inflammatory cytokines IL-1β and IL-6. These findings may represent the core molecular mechanism underlying the patient's fulminant, lethal disease course, as well as the rare manifestations of AKI and SCLS.

The possible mechanisms underlying this clinical presentation may include the following. ① Aberrant activation of the RAS-MAPK signaling pathway, along with excessive release of inflammatory cytokines, induces occludin phosphorylation and downregulates its total protein expression, while also reducing the expression of claudin family proteins. Collectively, these changes promote redistribution of tight junction proteins on the cell membrane, leading to disruption of vascular endothelial barrier integrity and increased permeability-an effect that can be blocked by specific MAPK inhibitors (29). The consequent leakage of plasma proteins and fluid results in hypoproteinemia, generalized edema, and polyserous effusions, ultimately culminating in SCLS (30, 31). ② The reduction in effective circulating volume caused by SCLS leads to hypotension and systemic hypoperfusion, thereby inducing AKI (32). Concurrently, the impairment of fluid excretion resulting from AKI complicates volume management in SCLS, forming a vicious cycle that ultimately contributes to irreversible outcomes (33). In parallel, activation of the RAS-MAPK pathway is involved in mediating renal tubular epithelial cell apoptosis, further promoting AKI progression (34). In renal tissue from patients with lupus nephritis, the expression of RAS-MAPK pathway-related genes (including NRAS, KRAS, *RIT1*, MRAS, SOS2, and MAP2K1) is significantly upregulated, whereas the expression of negative regulators (such as CBL, LZTR1, and NF1) is downregulated, confirming an aberrantly activated state of this pathway in kidney tissues (34). The interplay of these two mechanisms synergistically drives the initiation and progression of AKI, further substantiating that aberrant activation of the RAS-MAPK pathway represents a core mechanism underlying kidney injury in RASopathies (34). ③Structural disruption of the lymphatic return pathway, accompanied by continuous loss of proteins, fats, and lymphocytes, can rapidly lead to severe dehydration, hypoproteinemia, electrolyte imbalances, and systemic failure (35, 36). Meanwhile, a large PDA causes multi-organ hypoperfusion (e.g., in the intestines and myocardium) due to diastolic steal from the systemic circulation, resulting in ischemic and hypoxic tissue damage (37). In the present case, fluid imbalance resulting from refractory chylothorax and hemodynamic disturbances induced by a large PDA jointly amplified the synergistic effects of lymphatic-vascular injury, ultimately leading to irreversible progression of multiple organ dysfunction and death.

This case suggests that *RIT1*-NS should be highly suspected in neonates presenting with SCLS, chylothorax, and AKI, along with features such as high birth weight, polyhydramnios, and congenital heart disease, and WES is recommended for early diagnosis. Nevertheless, as a single-case report with *in vitro* data from rat cells, our findings have limited generalizability and do not prove causality; the absence of patient-tissue validation and confirmatory imaging/coagulation studies further limits exclusion of alternative causes. Future patient derived or animal models are needed to confirm direct pathogenicity.

## 5 Conclusion

The *RIT1:*c.247A>C p.(Thr83Pro) variant appears to aberrantly activate the RAS-MAPK pathway, which may contribute to lymphatic dysplasia and vascular endothelial injury. These changes might, in turn, be associated with the atypical manifestations of AKI and SCLS observed in this neonate with Noonan syndrome, and could potentially lead to multiorgan failure. The triad of SCLS, chylothorax, and AKI may serve as a useful clinical clue for suspecting *RIT1*-NS in critically ill neonates, and early genetic testing may be of value for prognostic assessment.

## Data Availability

The datasets presented in this study can be found in online repositories. The names of the repository/repositories and accession number(s) can be found in the article/Supplementary Material.

## References

[1] Romano AA Allanson JE Dahlgren J Gelb BD Hall B Pierpont ME. Noonan syndrome: clinical features, diagnosis, and management guidelines. Pediatrics. (2010) 126(4):746–59. 10.1542/peds.2009-3207 20876176

[2] Zenker M. Clinical overview on RASopathies. Am J Med Genet C Semin Med Genet. (2022) 190(4):414–24. 10.1002/ajmg.c.32015 36428239

[3] Hebron KE Hernandez ER Yohe ME. The RASopathies: from pathogenetics to therapeutics. Dis Model Mech. (2022) 15(2):dmm049107. 10.1242/dmm.049107 35178568 PMC8862741

[4] Carcavilla A Suárez-Ortega L Rodríguez Sánchez A Gonzalez-Casado I Ramón-Krauel M Labarta JI. Síndrome de Noonan: actualización genética, clínica y de opciones terapéuticas [Noonan syndrome: genetic and clinical update and treatment options]. An Pediatr (Engl Ed). (2020) 93(1):61.e1–4; Spanish. 10.1016/j.anpedi.2020.04.008 32493603

[5] Milosavljević D Overwater E Tamminga S de Boer K Elting MW van Hoorn ME. Two cases of *RIT1* associated Noonan syndrome: further delineation of the clinical phenotype and review of the literature. Am J Med Genet A. (2016) 170(7):1874–80. 10.1002/ajmg.a.37657 27109146

[6] Aoki Y Niihori T Banjo T Okamoto N Mizuno S Kurosawa K. Gain-of-function mutations in *RIT1* cause Noonan syndrome, a RAS/MAPK pathway syndrome. Am J Hum Genet. (2013) 93(1):173–80. 10.1016/j.ajhg.2013.05.021 23791108 PMC3710767

[7] Qiu Z Chang WT Chou YC Wen KC Ziying Y Yuen K. Prenatal case of *RIT1* mutation associated Noonan syndrome by whole exome sequencing (WES) and review of the literature. Taiwan J Obstet Gynecol. (2022) 61(3):535–8. 10.1016/j.tjog.2022.03.025 35595454

[8] Tafazoli A Eshraghi P Koleti ZK Abbaszadegan M. Noonan syndrome—a new survey. Arch Med Sci. (2017) 13(1):215–22. 10.5114/aoms.2017.64720 28144274 PMC5206377

[9] Richards S Aziz N Bale S Bick D Das S Gastier-Foster J. Standards and guidelines for the interpretation of sequence variants: a joint consensus recommendation of the American college of medical genetics and genomics and the association for molecular pathology. Genet Med. (2015) 17(5):405–24. 10.1038/gim.2015.30 25741868 PMC4544753

[10] Gos M Fahiminiya S Poznański J Klapecki J Obersztyn E Piotrowicz M. Contribution of *RIT1* mutations to the pathogenesis of Noonan syndrome: four new cases and further evidence of heterogeneity. Am J Med Genet A. (2014) 164A(9):2310–6. 10.1002/ajmg.a.36646 24939608

[11] Bertola DR Yamamoto GL Almeida TF Buscarilli M Jorge AA Malaquias AC. Further evidence of the importance of *RIT1* in Noonan syndrome. Am J Med Genet A. (2014) 164A(11):2952–7. 10.1002/ajmg.a.36722 25124994

[12] Koenighofer M Hung CY McCauley JL Dallman J Back EJ Mihalek I. Mutations in *RIT1* cause Noonan syndrome—additional functional evidence and expanding the clinical phenotype. Clin Genet. (2016) 89(3):359–66. 10.1111/cge.12608 25959749 PMC4760689

[13] Kouz K Lissewski C Spranger S Mitter D Riess A Lopez-Gonzalez V. Genotype and phenotype in patients with Noonan syndrome and a *RIT1* mutation. Genet Med. (2016) 18(12):1226–34. 10.1038/gim.2016.32 27101134

[14] Ramond F Duband S Croisille P Cavé H Teyssier G Adouard V. Expanding the cardiac spectrum of Noonan syndrome with *RIT1* variant: left main coronary artery atresia causing sudden death. Eur J Med Genet. (2017) 60(6):299–302. 10.1016/j.ejmg.2017.03.009 28347726

[15] Aly SA Boyer KM Muller BA Marini D Jones CH Nguyen HH. Complicated ventricular arrhythmia and hematologic myeloproliferative disorder in *RIT1*-associated Noonan syndrome: expanding the phenotype and review of the literature. Mol Genet Genomic Med. (2020) 8(7):e1253. 10.1002/mgg3.1253 32396283 PMC7336743

[16] Chen H Li X Liu X Wang J Zhang Z Wu J. Clinical and mutation profile of pediatric patients with RASopathy-associated hypertrophic cardiomyopathy: results from a Chinese cohort. Orphanet J Rare Dis. (2019) 14(1):29. 10.1186/s13023-019-1010-z 30732632 PMC6367752

[17] Suzuki K Wakamatsu M Ito Y Ishikawa M Shimotakahara A Futagawa H. Myeloproliferative disorder in a patient with *RIT1*-associated Noonan syndrome: case report and literature review. Pediatr Blood Cancer. (2024) 71(2):e30780. 10.1002/pbc.30780 38015090

[18] Zha P Kong Y Wang L Wang Y Qing Q Dai L. Noonan syndrome caused by *RIT1* gene mutation: a case report and literature review. Front Pediatr. (2022) 10:934808. 10.3389/fped.2022.934808 36160792 PMC9490085

[19] Aniol CV Prokop JW Rajasekaran S Pageau S Elizer SK VanSickle EA. Dilated coronary arteries in a 2-month-old with *RIT1*-associated Noonan syndrome: a case report. BMC Pediatr. (2023) 23(1):1. 10.1186/s12887-022-03818-w 36593444 PMC9806447

[20] Nakajima Y Watanabe Y Iwata K Yamada Y Higuchi S Mori J. Efficacy of sirolimus in treating refractory lymphatic malformation in Noonan syndrome: a case study. JCEM Case Rep. (2025) 3(6):luaf088. 10.1210/jcemcr/luaf088 40270997 PMC12015159

[21] Yaoita M Niihori T Mizuno S Okamoto N Hayashi S Watanabe A. Spectrum of mutations and genotype-phenotype analysis in Noonan syndrome patients with *RIT1* mutations. Hum Genet. (2016) 135(2):209–22. 10.1007/s00439-015-1627-5 26714497

[22] Van R Cuevas-Navarro A Castel P McCormick F. The molecular functions of *RIT1* and its contribution to human disease. Biochem J. (2020) 477(15):2755–70. 10.1042/BCJ20200442 32766847 PMC7787054

[23] Ichise T Yoshida N Ichise H. Ras/MAPK signaling modulates VEGFR-3 expression through Ets-mediated p300 recruitment and histone acetylation on the Vegfr3 gene in lymphatic endothelial cells. PLoS One. (2012) 7(12):e51639. 10.1371/journal.pone.0051639 23284731 PMC3524184

[24] Cuevas-Navarro A Wagner M Van R Swain M Mo S Columbus J. RAS-dependent RAF-MAPK hyperactivation by pathogenic *RIT1* is a therapeutic target in Noonan syndrome-associated cardiac hypertrophy. Sci Adv. (2023) 9(28):eadf4766. 10.1126/sciadv.adf4766 37450595 PMC10348673

[25] Meyer Zum Büschenfelde U Brandenstein LI von Elsner L Flato K Holling T Zenker M. *RIT1* Controls actin dynamics via complex formation with RAC1/CDC42 and PAK1. PLoS Genet. (2018) 14(5):e1007370. 10.1371/journal.pgen.1007370 29734338 PMC5937737

[26] Karkkainen MJ Ferrell RE Lawrence EC Kimak MA Levinson KL McTigue MA. Missense mutations interfere with VEGFR-3 signalling in primary lymphoedema. Nat Genet. (2000) 25(2):153–9. 10.1038/75997 10835628

[27] Sleutjes J Kleimeier L Leenders E Klein W Draaisma J. Lymphatic abnormalities in noonan syndrome Spectrum disorders: a systematic review. Mol Syndromol. (2022) 13(1):1–11. 10.1159/000517605 35221870 PMC8832235

[28] Gelb BD Yohe ME Wolf C Andelfinger G. New prospectives on treatment opportunities in RASopathies. Am J Med Genet C Semin Med Genet. (2022) 190(4):541–60. 10.1002/ajmg.c.32024 36533679 PMC10150944

[29] Kevil CG Oshima T Alexander B Coe LL Alexander JS. H(2)O(2)-mediated permeability: role of MAPK and occludin. Am J Physiol Cell Physiol. (2000) 279(1):C21–30. 10.1152/ajpcell.2000.279.1.C21 10898713

[30] Druey KM Greipp PR. Narrative review: the systemic capillary leak syndrome. Ann Intern Med. (2010) 153(2):90–8. 10.7326/0003-4819-153-2-201007200-00005 20643990 PMC3017349

[31] Druey KM Parikh SM. Idiopathic systemic capillary leak syndrome (clarkson disease). J Allergy Clin Immunol. (2017) 140(3):663–70. 10.1016/j.jaci.2016.10.042 28012935 PMC5481509

[32] Miyazawa R Nakamura H Kumagai M Anayama M Makino Y Nishikawa M. A case of frequent of prerenal acute kidney injury attacks: importance of recognizing systemic capillary leak syndrome: a case report. J Int Med Res. (2024) 52(11):3000605241301863. 10.1177/03000605241301863 39600049 PMC11603545

[33] Gatto S Di Maria A Bonesso C Vergone M Momesso E Scichilone LM. [Hypotension and generalized edema due to plasma leakage: a case report]. G Ital Nefrol. (2024) 41(5):2024-vol5; Italian. 10.69097/41-05-2024-07 39508706

[34] Bai Y Han G Guo K Yu L Du X Xu Y. Effect of lentiviral vector-mediated KSR1 gene silencing on the proliferation of renal tubular epithelial cells and expression of inflammatory factors in a rat model of ischemia/reperfusion injury. Acta Biochim Biophys Sin (Shanghai). (2018) 50(8):807–16. 10.1093/abbs/gmy071 30020400

[35] Yang MJ Kang S Hong SP Jin H Yoon JH Jin C. Cooperative ETS transcription factors are required for lymphatic endothelial cell integrity and resilience. J Clin Invest. (2025) 136(5):e196119. 10.1172/JCI196119 41460562 PMC13067931

[36] Kuwahara Y Tashiro H Takeshita G Egashira Y Maruyama A Ikeda Y. Refractory bilateral chylothorax and chylous ascites in a patient with systemic lupus erythematosus treated by pleuro-peritoneal and peritoneal-venous shunts along with cell-free and concentrated ascites re-infusion therapy. Respir Investig. (2024) 62(6):1191–4. 10.1016/j.resinv.2024.10.006 39442268

[37] Crystal MA Yacouby S Petit CJ. Ischemic changes associated with a large patent arterial duct in small infants. Catheter Cardiovasc Interv. (2014) 83(1):95–8. 10.1002/ccd.24639 22936526

